# Enhanced Genomic and Transcriptomic Resources for *Trichinella pseudospiralis* and *T. spiralis* to Underpin the Discovery of Molecular Differences between Stages and Species

**DOI:** 10.3390/ijms25137366

**Published:** 2024-07-05

**Authors:** Pasi K. Korhonen, Giuseppe La Rosa, Sunita B. Sumanam, Maria Angeles Gomez Morales, Alessandra Ludovisi, Edoardo Pozio, Daniele Tonanzi, Bill C. H. Chang, Neil D. Young, Robin B. Gasser

**Affiliations:** 1 Department of Veterinary Biosciences, Melbourne Veterinary School, Faculty of Science, The University of Melbourne, Parkville, VIC 3010, Australiasunita.sumanam@unimelb.edu.au (S.B.S.); billc@unimelb.edu.au (B.C.H.C.); nyoung@unimelb.edu.au (N.D.Y.); 2 European Union Reference Laboratory for Parasites, Department of Infectious Diseases, Istituto Superiore di Sanità, Viale Regina Elena 299, 00161 Rome, Italymariaangeles.gomezmorales@iss.it (M.A.G.M.); alessandra.ludovisi@iss.it (A.L.); edoardo.pozio@iss.it (E.P.); daniele.tonanzi@iss.it (D.T.)

**Keywords:** *Trichinella*, genome, transcriptomic resources, excretory/secretory (ES) proteins, host–parasite interactions, trichinellosis

## Abstract

Nematodes of the genus *Trichinella* are important pathogens of humans and animals. This study aimed to enhance the genomic and transcriptomic resources for *T. pseudospiralis* (non-encapsulated phenotype) and *T. spiralis* (encapsulated phenotype) and to explore transcriptional profiles. First, we improved the assemblies of the genomes of *T. pseudospiralis* (code ISS13) and *T. spiralis* (code ISS534), achieving genome sizes of 56.6 Mb (320 scaffolds, and an N50 of 1.02 Mb) and 63.5 Mb (568 scaffolds, and an N50 value of 0.44 Mb), respectively. Then, for each species, we produced RNA sequence data for three key developmental stages (first-stage muscle larvae [L1s], adults, and newborn larvae [NBLs]; three replicates for each stage), analysed differential transcription between stages, and explored enriched pathways and processes between species. Stage-specific upregulation was linked to cellular processes, metabolism, and host–parasite interactions, and pathway enrichment analysis showed distinctive biological processes and cellular localisations between species. Indeed, the secreted molecules calmodulin, calreticulin, and calsyntenin—with possible roles in modulating host immune responses and facilitating parasite survival—were unique to *T. pseudospiralis* and not detected in *T. spiralis*. These insights into the molecular mechanisms of *Trichinella*–host interactions might offer possible avenues for developing new interventions against trichinellosis.

## 1. Introduction

Parasitic helminths cause substantial morbidity in billions of animals and humans worldwide, and have a major adverse impact on agricultural industries due to associated productivity and financial losses in animals and plants each year. Parasitic nematodes (roundworms) of the genus *Trichinella* can cause a disease called trichinellosis in humans, characterised by initial clinical signs including nausea, diarrhoea, vomiting, fatigue, fever, and abdominal discomfort, often followed by headaches, fevers, chills, cough, swelling of the face and eyes, aching joints, muscle pains, itchy skin, and/or constipation [1]. This foodborne disease is transmitted directly from host to host via the ingestion of meat containing first-stage larvae (L1s, or muscle larvae, MLs). Although trichinellosis is endemic in many parts of the world, the impact of this disease in humans relates principally to acute outbreaks following the consumption of infected, raw, or insufficiently cured meat products, with key examples reported from Argentina, China, Laos, Papua New Guinea, Romania, Slovakia, and Vietnam [2].

*Trichinella* is a complex of species and genotypes with a worldwide geographic distribution, including Africa, the Americas, Asia, Asia–Pacific, and Europe [2]. Although only two phenotypes of *Trichinella* (i.e., non-encapsulated and encapsulated) are morphologically distinguishable based on the presence/absence of a collagen capsule around individual larvae within muscle cells of infected animals, molecular-genetic and biochemical investigations have classified 10 distinct species and three genotypes which display extensive biological variability [3,4]. Currently, the ‘encapsulated’ group (infecting only mammals) includes *T. spiralis* (T1), *T. nativa* (T2), *T. britovi* (T3), *T. murrelli* (T5), *T. nelsoni* (T7), *T. patagoniensis* (T12), *T. chanchalensis* (T13), and the *Trichinella* genotypes T6, T8, and T9; and the ‘non-encapsulated’ group (infecting birds and mammals or reptiles and mammals) includes *T. pseudospiralis* (T4), *T. papuae* (T10), and *T. zimbabwensis* (T11). These species or genotypes are often endemic to particular countries or regions, and some (e.g., *T. nativa* and *T. pseudospiralis*) can display quite significant degrees of intraspecific genetic variability, host usage, transmission patterns, and/or dispersal abilities [3,5,6,7].

The extensive genetic variability within the *Trichinella* complex is of major biological significance and interest, and can reflect divergent host–parasite relationships, epidemiology, and ecology [3,5,7]. Despite advances in our understanding of trichinellosis/*Trichinella*, there are still significant gaps in our knowledge of the molecular biology and biochemistry of various members of the *Trichinella* complex as well as host–parasite interactions at the molecular level [3]. Although most previous molecular studies have been informative, some of them were constrained by techniques or tools available at the time of study and relatively small data sets, limiting interpretations and conclusions.

Major developments in genomic, transcriptomic, proteomic, and bioinformatic technologies [8,9,10,11,12,13] provide unique opportunities to circumvent some of these challenges and limitations, and enable research on *Trichinella*, trichinellosis, and related areas. Indeed, relatively high-quality draft genomes exist for selected species/genotypes, including *T. spiralis* and *T. murrelli* [14,15,16,17], but there has been limited detailed study of transcriptomic and proteomic data sets representing distinct developmental stages of these taxa, to understand molecular alterations/variations occurring between such life cycle stages within individual species, and also molecular differences between species, particularly those that represent respective non-encapsulated and encapsulated phenotypes of *Trichinella*. 

Here, our goals were (i) to markedly enhance genomic and transcriptomic resources for selected representatives—*T. pseudospiralis* (non-encapsulated; code ISS13) and *T. spiralis* (encapsulated; code ISS534); (ii) to characterise and compare the composition of the transcriptomes of these two representatives; (iii) to study transcriptional variation between or among selected developmental stages of each of these species, and link these differences to respective stage-specific biological pathways or processes; and (iv) to explore transcriptional differences at key points of the life cycle between these representative non-encapsulated and encapsulated species.

## 2. Results

### 2.1. Creating Enhanced Transcriptomic Resources for T. pseudospiralis and T. spiralis

#### 2.1.1. Nucleic Acid Sequence Data Sets

For *T. pseudospiralis* and *T. spiralis*, we obtained totals of 5.4 Gb (N50: 12,722) and 6.0 Gb (N50: 12,514) genomic (PacBio) reads, and ~2.1 Gb of sequence data for each species after pre-processing (N50: 14,547 and 15,512, respectively). We also obtained 56,353,481 and 60,167,363 paired-end (Illumina, San Diego, CA, USA) short reads, 42,916,123 and 49,311,808 of which were retained after trimming and quality filtering (Table S1) for subsequent genome polishing. In addition, we obtained 247 Gb (*T. pseudospiralis*) and 240 Gb (*T. spiralis*) paired-end reads representing transcripts of NBL, L1 and adult stages, of which 164 Gb and 170 Gb were retained following trimming (Table S1). 

#### 2.1.2. Predicted Genes, Functional Annotation, and Comparison of Inferred Proteomes

For *T. pseudospiralis* and *T. spiralis*, we predicted 9495 and 10,485 genes encoding 10,773 and 12,588 proteins, respectively (Table 1). For *T. pseudospiralis*, 2100 of 3131 (67.1%) nematode Benchmarking Universal Single Copy Orthologs (BUSCOs) were identified (Table 1), 2060 of which (65.8%; comprising 1785 single-copy and 274 duplicated BUSCOs) were complete, with 40 (1.3%) being fragmented (Table 1). We inferred annotations for 6399 (67.4%) genes based on information from one or more of the four databases Pfam (*n* = 5210; 54.9%), PANTHER (*n* = 6026; 63.5%), SUPERFAMILY (*n* = 4524; 47.6%), and InterPro (*n* = 6154; 64.8%); and annotations for 9223 (97.1%) genes based on BLAST matches to *Trichinella* species (Table S2). Most genes (*n* = 7864; 82.8%) had BLAST matches in the KEGG database (Table S3), of which 3692 (46.9%) were assigned KEGG orthology terms. Assigned terms were linked to 42 protein groups/functions (KEGG BRITE; Table S3), most of which were in the categories “membrane trafficking” (*n* = 471), “chromosome and associated proteins” (*n* = 367), “exosome” (*n* = 218), “messenger RNA biogenesis” (*n* = 196), “transcription factors” (*n* = 193) and “ubiquitin system” (*n* = 192). For KEGG pathway modules, most of these terms were linked to “translation—RNA transport” (*n* = 142), “transport and catabolism—lysosome” (*n* = 139), “environmental adaptation—thermogenesis” (*n* = 138), “transcription—spliceosome” (*n* = 133), “folding sorting and degradation—protein processing in endoplasmic reticulum” (*n* = 124) and “translation—ribosome” (*n* = 113) relating to a total of 285 distinct pathways (Table S3). Sub-cellular localisations were inferred for 890 sequences predicted to have a signal peptide, with extracellular (*n* = 566; 63.5%), cell membrane (*n* = 151; 16.9%), and endoplasmic reticulum (*n* = 54; 6.1%) being predominant (Table S4). Overall, 7865 (73.0%) transcripts/proteins were annotated, and 2944 (28.1%) were hypothetical proteins (Table S5), 2606 of which had a BLASTp hit with a known, inferred *Trichinella* protein.

For *T. spiralis*, 2095 of 3131 (66.8%) nematode BUSCOs were identified (Table 1), 2051 of which (65.5%; comprising 1407 single-copy and 644 duplicated BUSCOs) were complete and 44 (1.4%) fragmented (Table 1). We inferred annotations for 6755 (64.4%) genes based on information from one or more of the four databases Pfam (*n* = 5414; 51.6%), PANTHER (*n* = 6352; 60.6%), SUPERFAMILY (*n* = 4669; 44.5%), and InterPro (*n* = 6469; 61.7%) as well as annotations for 10,080 (96.1%) genes based on BLAST hits to *Trichinella* species (Table S6). Most genes (*n* = 9059; 86.4%) had BLAST matches to KEGG database (Table S6), of which 3666 (40.5%) were assigned to KEGG orthology terms. Assigned terms were linked to 42 protein groups/functions (KEGG BRITE; Table S3), most of which were assigned to the categories “membrane trafficking” (*n* = 481), “chromosome and associated proteins” (*n* = 361), “exosome” (*n* = 225), “messenger RNA biogenesis” (*n* = 200), “spliceosome” (*n* = 197), and “transcription factors” (*n* = 193). In KEGG pathway modules, most terms were assigned to “transport and catabolism—lysosome” (*n* = 182), “environmental adaptation—thermogenesis” (*n* = 167), “transcription—spliceosome” (*n* = 160), “folding sorting and degradation—protein processing in endoplasmic reticulum” (*n* = 149), “translation—RNA transport” (*n* = 132), and “transport and catabolism—endocytosis” (*n* = 131) for a total of 284 distinct pathways (Table S3). Sub-cellular localisations were inferred for 869 sequences predicted to have a signal peptide, with “extracellular” (*n* = 552; 63.5%), “cell membrane” (*n* = 149; 17.1%), and “endoplasmic reticulum” (*n* = 51; 5.9%) being predominant (Table S7). Overall, 8673 (68.9%) transcripts/proteins were annotated, and 3949 (31.4%) were hypothetical proteins (Table S8), of which 2904 had a BLASTp hit to a hypothetical *Trichinella* protein.

A comparison of protein sequences (*n* = 10,773 and 12,588) inferred from the transcriptome of *T. pseudospiralis* with those of *T. spiralis* identified 8815 (81.7%) *versus* 10,179 (80.6%) orthologous sequences, respectively, 4450 of which represented one-to-one (‘single-copy’) orthologs (Table S9). In total, 1981 (18.4%) of all protein sequences inferred for *T. pseudospiralis* had no ortholog in *T. spiralis*. Conversely, 2443 (19.4%) of all protein sequences for *T. spiralis* had no ortholog in *T. pseudospiralis*.

#### 2.1.3. Enriched Biological Pathways

**Figure 1:**
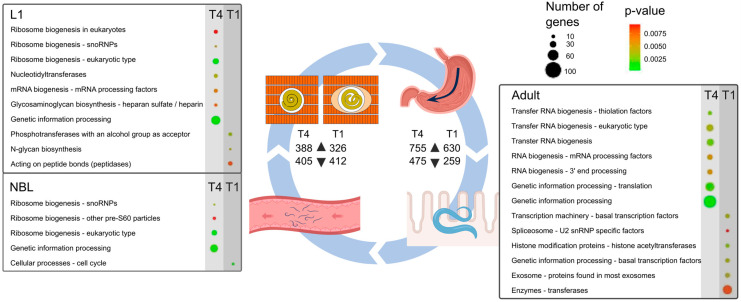
The diagram represents the *Trichinella* life cycle, in which first-stage larvae (L1) within striated muscle are ingested and then released in the stomach, progress through to the small intestine, enter the epithelium of the small intestine, develop to fourth-stage larvae and then to the adult stage (within 48 h), copulate, adult males die and adult females lay newborn larvae (NBLs) into lacteals and capillaries, after which individual larvae enter and establish within striated muscle cells. The tables show KEGG BRITE gene family enrichments in NBL, L1, and adult stages between *Trichinella pseudospiralis* (T4) and *T. spiralis* (T1). *p*-values are colour-coded in circles from green to red (low to high); the size of a circle indicates the number of genes linked to an enriched pathway or process. For each species, the numbers of “up-regulated” (▲) and “down-regulated” (▼) transcripts are indicated.

We investigated transcriptional differences between developmental stages, and focused our attention on upregulated transcripts from NBL to L1 and from L1 to adult developmental stages, and then assigned these transcripts to KEGG pathways. For *T. pseudospiralis*, transcripts upregulated from NBL to L1 (*n* = 388; Figure 1) associated with the metabolism of amino acids and lipids, xenobiotic biodegradation, cellular processes involving lysosomes, and cell growth and death related to apoptosis (Table S10); transcripts upregulated from L1 to adult (*n* = 755; Figure 1) linked to cellular processes, including cell growth and death related to meiosis, cell cycle and cellular senescence, focal adhesion, and the proteasome and lysosome systems (Table S10). For *T. spiralis*, transcripts upregulated from NBL to L1 (*n* = 326; Figure 1) related to the metabolism of purine nucleotides and, as for *T. pseudospiralis*, the metabolism of amino acid and lipids, xenobiotic biodegradation, in cellular processes linked to lysosomes as well as cell growth and death related to apoptosis (Table S11); from L1 to the adult stage, upregulated transcripts (*n* = 630; Figure 1) were predominantly linked to cellular processes and cell growth and death related to meiosis, cell cycle and cellular senescence, focal adhesion and lysosome, like in *T. spiralis*, and a small number (*n* = 14) were assigned to the Hippo signalling pathway (Table S11).

#### 2.1.4. Protein Groups Inferred to Be Involved in Parasite–Host Interplay

Subsequently, we inferred key protein groups in *T. pseudospiralis* and *T. spiralis* with likely or proposed roles in host–parasite interactions and with signal peptides—supported by previously published evidence (Tables S12 and S13).

**Figure 2:**
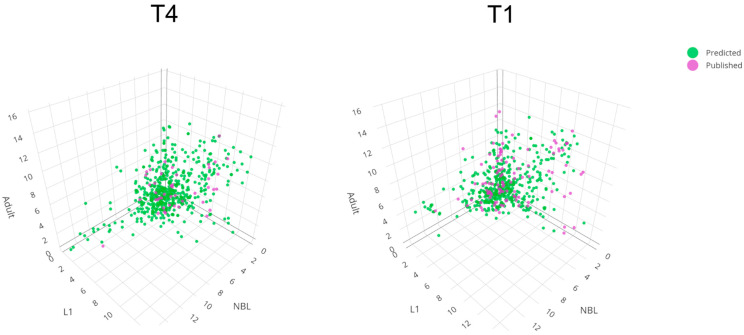
Three-dimensional display of transcription levels (log_2_ TPM) of genes predicted to encode excretory/secretory (ES) proteins in newborn larvae (NBL), first-stage larvae (L1s), and adult stages of *Trichinella pseudospiralis* (T4, **left**) and of *T. spiralis* (T1, **right**) in the present study (green). For comparison, transcription levels of genes known to encode ES proteins from published studies (pink). Detailed results are presented in Tables S12 and S13, respectively.

For *T. pseudospiralis*, we linked 36 isoforms, with high confidence (Levenshtein distance of ≤0.50), to ES protein sequences (cf. Table S12; Figure 2). These proteins included 45 kDa antigens (likely associated with immune modulation and invasion of host tissues), 5′ nucleotidases (inferred to prevent platelet aggregation) [18], an SCP/TAPS protein, Dnase II enzymes, serine/threonine-protein kinase, serine proteases and other peptidases (likely involved in the degradation of host tissues for migration and feeding, and immune modulation) [19,20] (Table S12). Interestingly, the molecules calmodulin, calreticulin, and calsyntenin (TPS_02755_1s, TPS_02704_2s and TPS_03873_1s, respectively; Table S12)—identified and highly transcribed in all developmental stages—might have a role in regulating calcium homeostasis in host cells [21]. In addition, a peptidase inhibitor (TPS_09167_1s; Table S12) was highly transcribed in NBL. There was a tendency for fewer ES protein genes to be transcribed in the NBL stage compared with the L1 and adult stages. This was evidenced by 19 of a total of 36 transcripts inferred to encode ES proteins being specifically downregulated in the NBL stage, contrasting with a single upregulated transcript (Table S12). This bias in numbers may relate to most published studies focusing on investigating the L1 stage (rather than the NBL and adult stages, which are much more challenging to yield for experimentation). This proposal is supported by the identification of a total of 891 transcripts predicted to encode ES proteins in all three stages of *T. pseudospiralis* studied here, 151 of which were shown to be differentially downregulated and 94 upregulated in the NBL stage (Table S4).

Based on published evidence [22,23,24,25,26,27,28], *T. spiralis* secretes a multitude of proteins that likely manipulate or modulate the host environment, of which we confidently mapped most to predicted isoforms (*n* = 91; normalised Levenshtein distance of ≤0.50; Table S13; Figure 2). These transcripts include three that encode 53-kDa glycoproteins (which are immune-modulatory to T cells, macrophages, and cytokines in the host animal) [29,30], 5′-nucleotidases (which prevent platelet aggregation) [18], Dnase II enzymes, serine proteases (involved in the degradation of host tissues for migration and/or feeding, and possible immune modulation) [19,20], cathepsins (with possible roles in intracellular protein degradation, energy metabolism, and/or immune modulation), ADP-ribose pyrophosphatase (likely involved in DNA repair and other cellular processes including transcription and modulation of chromatin structure), and galectins (which may contribute to tissue invasion and immune evasion through extracellular matrix degradation and/or the modulation of host immune cell function) [31] (Table S13). Unlike in *T. pseudospiralis*, respective transcripts encoding calmodulin, calreticulin, and calsyntenin were not detected in any of the three stages of *T. spiralis* studied here, and transcription of the gene encoding a peptidase inhibitor (TSP_07690_1s) was very low (Table S7). Similar to *T. pseudospiralis*, there was a tendency for fewer transcripts encoding ES proteins in the NBL stage as compared with the L1 and adult stages. This was evidenced by 34 of a total of 91 transcripts inferred to encode ES proteins being downregulated in the NBL stage. By contrast, only six of these transcripts were differentially upregulated (Table S13). Again, such a bias might arise from previous studies focusing on studying the L1 stage (rather than the NBL and adult stages, which are much more challenging to yield for experiments). This latter proposal is supported by the inference of a total of 869 transcripts encoding ES proteins, 100 of which were differentially downregulated and 86 upregulated in the NBL stage (Table S7).

#### 2.1.5. Cellular Localisations of Hypothetical Proteins

Of all 2944 hypothetical proteins (length range: 29–2089 amino acids (aa); mean: 183 aa, median: 118 aa) inferred for *T. pseudospiralis*, 2606 (88.9%) had an ortholog in other *Trichinella* species; all but 12 transcripts were full-length, each with start and stop codons, thus representing *bona fide* transcripts (Table S5). This result compares with 3949 hypothetical proteins (length range: 29–4867 aa; mean: 238 aa, median: 140 aa) for *T. spiralis*, of which 2904 (73.5%) had an ortholog in *Trichinella* species; all but 67 transcripts were full-length, each with start and stop codons (Table S8). Most *T. pseudospiralis* proteins with signal peptides (encoded by 255 transcripts) were inferred to be extracellular (*n* = 203) or within the cell membrane (*n* = 23) (Table S4). In addition, most of the *T. spiralis* proteins with signal peptides (encoded by 280 transcripts) were inferred to be extracellular (n = 221) or in the cell membrane (*n* = 27) (Table S7).

### 2.2. Linking Transcription within Developmental Stages to Biological Pathways/Processes to Understand Each of the Two Trichinella Species Better at the Molecular Level

The development of members of the genus *Trichinella* involves a series of tightly-timed biological processes (Figure 1). Embryogenesis generates the basic tissue types of the nematode, and each tissue type differentiates at a specific point in the developmental cycle. Post-embryonic structures required for parasitism and reproduction then differentiate through the larval stages (L1, L3, and L4) to the adult stage. This includes the specialised development of tissues, sexual differentiation, and gametogenesis in the adult stage. Substantial growth occurs between the L1 and the dioecious adult stage. On the one hand, the development of the L1 stage within muscle cells is ‘arrested’, although larvae are still motile and can survive here for years. On the other hand, after a susceptible host ingests L1-infected muscle tissue, the L1 undergoes very rapid development (within a few hours for both *T. pseudospiralis* and *T. spiralis*) in the gut environment (following exposure to gastric/small intestinal juices) via short-lived L2 and L3 to the adult stage (Figure 1). Each of these stages has different requirements in terms of motility, sensory perception, metabolism, and the regulation of hormones of the endocrine system, and there are marked biological differences between some species and genotypes [3,32]. A key feature that distinguishes *T. pseudospiralis* from *T. spiralis* is the absence of a distinct collagen capsule at the L1 stage in the muscle cell [32], indicating molecular, biochemical, and/or physiological uniqueness.

For the L1 stage of *T. pseudospiralis*, pathway analysis revealed a specific enrichment for ribosome biogenesis (ko03009) linked to 19 genes, of which three (TPS_01253_1s, TPS_05940_1s, and TPS_01904_1s) linked to small nucleolar ribonucleoproteins (snoRNP); phosphotransferases (ko01000: nucleotidyl-transferases) linked to 5 DNA/RNA polymerase proteins (genes: TPS_08936_1s, TPS_07281_1s, TPS_04919_1s, TPS_07413_1s and TPS_00888_1s) and one to CCA tRNA nucleotidyl-transferase (TPS_04631_1s); and glycan biosynthesis and metabolism (ko00001) for glycosaminoglycan biosynthesis, including two sulfotransferases (TPS_03416_1s and TPS_05021_1s) (Table S14; Figure 1). This contrasted with the situation for the L1 stage of *T. spiralis*, in which hydrolases (ko01000), including members of the chymotrypsin family (S1), linked to three genes (TSP_01815_1s, TSP_02834_1s and TSP_03378_1s) which were enriched as well as a deubiquitinase (TSP_00103_1s) acting on carboxyl-terminal (Table S15; Figure 1).

For the adult stage of *T. pseudospiralis*, pathway analysis revealed a specific enrichment for genetic information processing (ko00001; translation) supported by 36 distinct genes; transfer RNA biogenesis (ko03016; 3′-processing and CCA adding factors; tRNA modification factors and thiolation factors) linked to 19 distinct genes; and messenger RNA biogenesis (ko03019; mRNA processing factors and 3′-end processing) also linked to seven genes (Table S14; Figure 1).

In contrast, pathways in the adult stage of *T. spiralis* were specifically enriched for chromosome- and associated proteins (ko03036; histone modification proteins, including histone acetyltransferases) linked specifically to four genes; transcription (ko00001) and transcription machinery (RNA polymerase; ko03021; RNA polymerase II system and basal transcription factors) linked to five genes; spliceosome (ko03041; U2 snRNP specific factors) linked to three genes and exosome (ko04147; proteins found in most exosomes) associated with six genes (Table S15; Figure 1).

Conspicuous in the NBL stage of *T. pseudospiralis* was the enrichment for ribosome biogenesis (ko03009), including snoRNPs linked to three genes and pre-60S particles associated with five genes; and genetic information processing (ko00001) linked to 47 genes (Table S14; Figure 1), contrasting the NBL stage of *T. spiralis* which was enriched solely for molecules encoded by four genes involved in cellular processes (ko00001), including cell growth/death and cell cycle (Table S15; Figure 1).

## 3. Discussion

The contiguity of the present genome assembly for *T. pseudospiralis* (ISS13) with 320 contigs (N50 = 1,024,593 bp; L50 = 16; Table 1) is a significant improvement compared to the previous genome assembled to 7221 scaffolds (N50 = 235,426; L50 = 51) using short reads [17]. BUSCO results improved (from 65.3% to 65.8%) and repeat content increased from 22.3% to 25.5% in *T. pseudospiralis*, as expected for a more contiguous genome. The number of protein-encoding genes estimated here from the contiguous genome was smaller than for a previous draft genome (Table 1) [17], because we required RNA-based evidence for gene prediction and also overcame a possible over-estimation in gene numbers that is inherent in predicting genes from a fragmented genome; also the use of Braker3 can underestimate the number of genes in favour of accuracy [33]. The number of predicted genes was also lower for the *T. spiralis* (ISS534) genome compared to that published first for this species (ISS195) (Table 1) [14]. Despite this, the BUSCO results for ISS534 (65.8%) and ISS195 (67.4%) were similar (Table 1). The difference in gene numbers and lengths relates to the requirement for RNA data as evidence for the prediction of genes. The relatively large numbers of duplicated orthologs (BUSCO) for both ISS13 and ISS534 relate to the isoforms predicted (Table 1). The large numbers of proteins predicted to be hypothetical in the genomes of both *Trichinella* genomes suggest their large evolutionary distance from better studied invertebrates (e.g., *C. elegans* and *D. melanogaster*) and the unique biology of *Trichinella* and associated gene functions. Interestingly, the majority of the hypothetical proteins predicted to be excretory/secretory were inferred to be extracellular, which provides an opportunity to explore their role(s) in modulating host responses. Exploring these proteins produced in the NBL stage would be particularly pertinent to understanding immune responses early in infection.

This is the first investigation of transcription in NBL and adult stages using RNA sequence data from biological replicates, allowing a confident analysis of transcription profiles for NBL, L1, and adult stages, and the identification of transcripts encoding proteins, such as calmodulin, calreticulin, and calsyntenin, that are unique to *T. pseudospiralis* to the exclusion of *T. spiralis*. 

Calmodulin is an intracellular Ca^2+^-sensor that has important roles in Ca^2+^-mediated signalling [34]. As calmodulin is known to influence host immune responses by modulating pathways that control immune cell activation and inflammatory responses, it might be involved in downregulating pro-inflammatory cytokines or in altering immune cell signalling to prevent a pronounced immune attack against parasites [35]. Calmodulin might facilitate the restructuring of host cells to create a unique niche for *T. pseudospiralis*, providing nutrients and protection from the host—as distinct from the nurse cell arrangement in *T. spiralis* [36]. This protein might also assist in the secretion of molecules that modulate the host environment via the regulation of vesicle trafficking [37], including the release of excretory/secretory products that alter host cell functions that benefit the parasite. As calmodulin’s role in calcium signalling is crucial for numerous cellular processes [34,35,38], it might ensure that calcium levels are maintained to support parasite survival; this is particularly important in muscle cells [35], where individual larvae reside and likely regulate calcium signalling to avoid muscle cell death and maintain a favourable habitat in these cells. Moreover, calmodulin might activate pathways specific to *T. pseudospiralis* that help mitigate oxidative damage [39], ensuring its survival, and regulate heat shock proteins to aid in managing crucial protein folding and preventing damage under stress conditions [34,35,40], which could be vital in maintaining the function and integrity of proteins within the host (including within muscle cells).

On the other hand, calreticulin, or calregulin, is a multifunctional, soluble calcium-binding chaperone protein primarily within the endoplasmic reticulum that is involved a range of cellular processes, such as cell adhesion [41], and might also enable the parasite’s movement through host tissues and entry into muscle cells by interacting with components of the extracellular matrix [42] and by modulating the adhesive properties of host cells. It likely plays a complementary role to calmodulin in modulating calcium homeostasis and protein folding [41], and in suppressing immune responses specific to *T. pseudospiralis*.

Also calsyntenin, representing transmembrane proteins (cadherin superfamily) that bind calcium [43,44], is likely involved in calcium homeostasis as well as the interaction with host cell adhesion molecules, facilitating parasite invasion and attachment to host cells. As calsyntenin is usually associated with neural functions in other contexts [43,45], its role in the host–parasite relationship may involve modulating host neuronal responses to enable parasite invasion, establishment, and survival. Calsyntenin is proposed to influence host neuronal responses, potentially modulating neuroimmune signalling pathways. By interacting with host neurons, calsyntenin might alter the release of neurotransmitters or neuroimmune factors [46], affecting the local immune environment and promoting parasite survival. This neuroimmune cross-talk may be responsible for a suppression of pro-inflammatory cytokines or the promotion of anti-inflammatory signalling, creating a favourable environment for *T. pseudospiralis*.

In addition to calmodulin, calreticulin, and calsyntenin, a gene encoding a hypothetical extracellular peptidase inhibitor (TPS_09167_1s; Table S12) was discovered to be highly transcribed specifically in NBLs of *T. pseudospiralis*, contrasting the conspicuous transcription of genes (TSP_10375_1s, TSP_10375_2s and TSP_10378_1s; Table S13) encoding proteins already known to be exclusively produced, *tyvelose*-decorated, and secreted by stichocytes of the L1 stage of *T. spiralis* and other encapsulated species of *Trichinella* into the milieu of the infected host muscle cell [36,47,48].

Taken together, the molecules identified here as specific to *T. pseudospiralis* (including calmodulin, calreticulin, calsyntenin, and the hypothetical peptidase inhibitor encoded by TPS_09167_1s) are proposed to orchestrate a number of critical processes or pathways that are specific to *T. pseudospiralis* and could be linked to unique aspects of the biology and morphology (i.e., the non-encapsulated phenotype) of this particular species. Understanding the roles of these molecules should offer insights into the molecular mechanisms of *T. pseudospiralis*–host interactions. In conclusion, the present investigation provides a stimulus for future molecular studies of the range of other species and genotypes of *Trichinella* (with ISS codes) employing advanced multi-omics and informatics, combined with in vitro and in vivo experimentation. The hope is that fundamental insights achieved via the use of genomic, transcriptomic, and proteomic approaches will enable the development of improved tools for the specific diagnosis and surveillance of trichinellosis (to a species and/or genotypic level) and the design of novel interventions, including effective anti-*Trichinella* vaccines and therapeutics.

## 4. Materials and Methods

### 4.1. Production and Procurement of L1s, Adults, and Newborn Larvae (NBLs) of Trichinella

*Trichinella pseudospiralis* (T4; code ISS13) and *T. spiralis* (T1; code ISS534) were maintained and produced at the International Trichinella Reference Center (https://trichinella.iss.it//; accessed on 12 May 2023) [49], Istituto Superiore di Sanita’ (ISS), Rome, Italy. L1s, adults, and NBLs of *T. pseudospiralis* and *Trichinella spiralis* were each produced in Wistar rats. L1s were recovered from host musculature by pepsin (1%)–HCl (1%) digestion at 40 °C for 30 min, sedimented, washed extensively in physiological saline, and then suspended in 90% ethanol for storage at −80 °C until subsequent nucleic acid isolation. Adult stages (both sexes) were isolated from the small intestine of Wistar rats 31 h after oral infection (via gavage) with L1s (*n* = 10,000 per rat); worms were washed in physiological saline, centrifuged (600× *g*), snap-frozen in liquid nitrogen, and stored at −80 °C until nucleic acid isolation. NBLs were collected from adult female worms from the small intestine 5 days following oral infection with L1s (*n* = 10,000 per rat); these females were gravid with embryos and larvae (i.e., pre-NBLs). Adult males and females were washed in physiological saline and incubated in saline plus antibiotics (final concentrations: 100 IU/mL of penicillin, 100 μg/mL of streptomycin and 0.25 μg/mL of amphotericin B) at 37 °C for 18 h. Viable NBLs were collected following migration through a filter (mesh size: 20 µm) and then concentrated by centrifugation, snap-frozen in liquid nitrogen, and stored at −80 °C or suspended in RNAlater^®^ (Invitrogen, Waltham, MA, USA) until RNA isolation.

### 4.2. Nucleic Acid Isolation and Sequencing

High molecular weight genomic DNA of *T. pseudospiralis* or *T. spiralis* was isolated from 100,000 pooled L1s using the Gentra^®^ Puregene^®^ Tissue Kit (Qiagen, Hilden, Germany). The total DNA amount was determined using a *Qubit* fluorometer dsDNA HS Kit (Invitrogen), and the DNA integrity was verified using a Bioanalyzer 2100 (Agilent, Santa Clara, CA, USA). Long-read sequencing of libraries constructed using the 20 kb Template Preparation employing the BluePippin™ Size-Selection System was conducted employing an established protocol (Pacific Biosciences [PacBio], Menlo Park, CA, USA) [50]. Short-read paired-end (PE) libraries (insert size: 100 bp) were constructed, assessed for quality and size distribution using the Bioanalyzer 2100, and then sequenced on the Illumina HiSeq 2500 platform.

Prior to RNA isolation, RNAlater^®^ was aspirated from samples, and worms were extensively washed in nuclease-free water. Then, total RNA was isolated from 3–4 (biological) replicate samples of L1s, adults, and NBLs (each sample containing ~ 30,000, 3000 and 30,000 worms, respectively) using the TRIzol™ reagent (cat. no. 15596026, Thermo Fischer Scientific Inc., Waltham, MA, USA) and treated with RNase-free TURBO DNase (Ambion^®^, cat no. AM1907, Thermo Fisher Scientific Inc., Waltham, MA, USA). The size, integrity, and concentration of RNA were estimated using a 4200 TapeStation System RNA ScreenTape Assay (Agilent Technologies, Waldbronn, Germany) and a Qubit 3.0 Flourometer RNA High Sensitivity Assay (Life Technologies, Carlsbad, CA, USA). The Illumina^®^ Stranded mRNA Prep Ligation kit (Illumina, San Diego, CA, USA) was used to construct stranded cDNA libraries (150 bp reads; paired-end) according to the manufacturer’s instructions and then sequenced on an Illumina HiSeq™ 4000 instrument. RNA-sequence data are publicly available via the National Center of Biotechnology Information (NCBI) Sequence Read Archive (SRA)—accession codes: SRR28903633 to SRR28903645; SRR28901789 to SRR28901803 (Table S1). 

### 4.3. Nuclear Genomes, Prediction of Repetitive Elements, Protein-Encoding Genes, and Functional Annotation

The nuclear genomes of *T. pseudospiralis* and *T. spiralis* were each assembled here from sequence reads (SRR28878465 and SRR28878466) produced by PacBio sequencing and then polished using Illumina sequence data (SRR28920121 and SRR28920122) using an established pipeline [51]. Genomic repeats specific to each *Trichinella* species were inferred using the program RepeatModeler v2.0.4 [52] and the Dfam v3.7 database [53]. Known transposons in the Dfam database as well as inferred custom repeats and simple repeats were masked in each assembly using RepeatMasker v4.1.5 [54]. The program Braker v3.0.3 [33] was used to predict genes in each respective, masked genome, employing pooled RNA-sequence data for all developmental stages (i.e., L1s, adults, and NBLs; 3–4 replicates per stage) of each species (Table S1). The RNA-sequences were first trimmed to achieve a minimum Phred quality score of 30 and a minimum length of 60 bp, and all adapter sequences were removed using Trimmomatic v0.36 [55]. The trimmed reads were then mapped to each respective, masked genome using the program HISAT-2 v2.1.0 [56]. The BAM file of the mapped RNA-sequence reads was then used as evidence (via Braker v3.0.3) for gene transcription. Genes were first assessed for quality using the program table2asn [57] (23 October 2023) from NCBI and BUSCO v5.1.2 [58]. Full-length genes were then annotated using InterPro v5.51-85.0 [59] and BLAST database UniProt/SwissProt [60] (14 September 2023), eukaryotes in KEGG [61] (14 May 2019) and NCBI NR [62] (4 February 2021), and proteomes of *T. spiralis* and *T. pseudospiralis* in WormBase (version WBP18; WormBase). Files for submission to NCBI were prepared using custom scripts and the program table2asn. Secreted proteins were inferred using the program SignalP v6.0 [63]. The sub-cellular location of protein sequences was predicted computationally using the program DeepLoc v2.0 [64] employing a (stringent) confidence cut-off score of 0.8. Excretory/secretory (ES) proteins (‘secretome’) inferred from each genome were compared against publicly available protein sequences (via NCBI) using BLASTp (E-value: 10^−8^), and normalised Levenshtein distances (≤0.50) [65] were recorded to assess the validity of BLAST hits. A protein was defined as ‘hypothetical’ if its sequence did not match any protein in any of the abovementioned public databases and did not have a functional annotation.

### 4.4. Differential Transcription Analysis

Differential transcription among key developmental stages (i.e., L1, adult, and NBL) of each *T. pseudospiralis* and *T. spiralis* was explored upon pairwise comparison using the program edgeR [66]. Additionally, differential transcription between *T. pseudospiralis* and *T. spiralis* was explored using the orthologous transcripts for each developmental stage. Orthologous transcripts were identified using the program OrthoMCL [67]. To validate the resultant syntenic blocks, at least five single copy orthologs (SCO) were required to define a syntenic block; other orthologous transcripts were then selected if they were located between these SCOs within the syntenic blocks. Available RNA sequence data used for gene prediction were mapped to predicted transcripts to infer expected read counts using the programs bowtie2 [68] and RSEM [69]. Differential transcripts were then inferred using a false discovery rate (FDR) of ≤0.001.

### 4.5. Pathway Enrichment Analysis

For each species of *Trichinella*, differential transcripts were subjected to enrichment analysis using Kyoto Encyclopedia of Genes and Genomes (KEGG) pathways and KEGG BRITE terms. Enriched KEGG pathways were inferred based on KEGG BLAST hits (E-value of <10^−8^) to KEGG Orthology (KO) terms [70]; KO terms were then mapped to the KEGG Orthology Based Annotation System (KOBAS) database [71] and enriched KEGG pathways and BRITE terms were then identified and assigned (*p*-value < 0.01; Fisher’s Exact test).

## Figures and Tables

**Table 1:** Features of *Trichinella pseudospiralis* and *T. spiralis* draft genomes.

Features (Parameters)	*T. pseudospiralis* (ISS13) This Study	*T. pseudospiralis* (ISS13) Ref. [17]	*T. spiralis* (ISS534) This Study	*T. spiralis* (ISS195) Ref. [14]
Genome size (bp)	56,636,606	49,162,916	63,452,358	63,525,422
Number of scaffolds	320	7221	568	6863
N50 (bp); L50	1,024,593; 16	235,426; 51	438,897; 39	6,373,445; 4
N90 (bp); L90	66,105; 83	60,440; 206	31,707; 264	2047; 919
Genome GC content (%)	32.6	32.6	33.6	33.9
Repetitive sequences (%)	25.3	22.3	27.7	25.6
Exonic proportion; incl. introns (%)	21.6; 45.9	35.7; 76.0	19.7; 43.7	22.2; 46.7
Number of putative coding genes; isoforms	9495; 10,773	12,659; 17,161	10,485; 12,588	16,380; 15,840
Mean; median gene length (bp)	2743; 1792	2950; 1241	2653; 1700	1817; 1078
Mean; median CDS length (bp)	1361; 951	1046; 522	1236; 897	955; 576
Mean exon number per gene	7.4	6.6	7.2	5.4
Mean; median exon length (bp)	175; 126	211; 131	166; 120	178; 129
Mean; median intron length (bp)	227; 73	280; 78	234; 72	198; 83
Coding GC content (%)	42.7	42.6	43.3	43.2
BUSCO complete; duplicated; fragmented	2060; 274; 40	2044; 785; 17	2051; 644; 44	2110; 74; 105
BUSCO completeness: complete; partial (%)	65.8; 67.1	65.3; 65.8	65.5; 66.9	67.4; 70.8

## Data Availability

Sequenced read data can be found at NCBI SRR with accession codes SRR28903633 to SRR28903645; SRR28901789 to SRR28901803 for RNA short read data, SRR28920121 and SRR28920122 for DNA short read data, and SRR28878465 and SRR28878466 for DNA long read data. This Whole Genome Shotgun project has been deposited in DDBJ/ENA/GenBank under the accessions JBEUSZ000000000 and JBEUSY000000000. The project data are available in the GenBank database through BioProject PRJNA1089635.

## Supplementary Materials

The following supporting information can be downloaded at: https://www.mdpi.com/article/10.3390/ijms25137366/s1.
